# Integrating deep learning for visual question answering in Agricultural Disease Diagnostics: Case Study of Wheat Rust

**DOI:** 10.1038/s41598-024-79793-2

**Published:** 2024-11-15

**Authors:** Akash Nanavaty, Rishikesh Sharma, Bhuman Pandita, Ojasva Goyal, Srinivas Rallapalli, Murari Mandal, Vaibhav Kumar Singh, Pratik Narang, Vinay Chamola

**Affiliations:** 1 Department of Computer Science and Information Systems, Birla Institute of Technology and Science, Pilani, Rajasthan, 333031 India; 2 Department of Civil Engineering, Birla Institute of Technology and Science, Pilani, Rajasthan, 333031 India; 3 School of Computer Engineering, KIIT Bhubaneshwar, Patia, India; 4 Division of Plant Pathology, ICAR-Indian Agricultural Research Institute, New Delhi, India; 5 Department of Electrical and Electronics Engineering, Birla Institute of Technology and Science, Pilani, Rajasthan, 333031 India

**Keywords:** Deep learning, Plant Disease, Visual question answering, Wheat rust, Engineering, Computer science, Scientific data, Software

## Abstract

This paper presents a novel approach to agricultural disease diagnostics through the integration of Deep Learning (DL) techniques with Visual Question Answering (VQA) systems, specifically targeting the detection of wheat rust. Wheat rust is a pervasive and destructive disease that significantly impacts wheat production worldwide. Traditional diagnostic methods often require expert knowledge and time-consuming processes, making rapid and accurate detection challenging. We drafted a new, WheatRustDL2024 dataset (7998 images of healthy and infected leaves) specifically designed for VQA in the context of wheat rust detection and utilized it to retrieve the initial weights on the federated learning server. This dataset comprises high-resolution images of wheat plants, annotated with detailed questions and answers pertaining to the presence, type, and severity of rust infections. Our dataset also contains images collected from various sources and successfully highlights a wide range of conditions (different lighting, obstructions in the image, etc.) in which a wheat image may be taken, therefore making a generalized universally applicable model. The trained model was federated using Flower. Following extensive analysis, the chosen central model was ResNet. Our fine-tuned ResNet achieved an accuracy of 97.69% on the existing data. We also implemented the BLIP (Bootstrapping Language-Image Pre-training) methods that enable the model to understand complex visual and textual inputs, thereby improving the accuracy and relevance of the generated answers. The dual attention mechanism, combined with BLIP techniques, allows the model to simultaneously focus on relevant image regions and pertinent parts of the questions. We also created a custom dataset (WheatRustVQA) with our augmented dataset containing 1800 augmented images and their associated question-answer pairs. The model fetches an answer with an average BLEU score of 0.6235 on our testing partition of the dataset. This federated model is lightweight and can be seamlessly integrated into mobile phones, drones, etc. without any hardware requirement. Our results indicate that integrating deep learning with VQA for agricultural disease diagnostics not only accelerates the detection process but also reduces dependency on human experts, making it a valuable tool for farmers and agricultural professionals. This approach holds promise for broader applications in plant pathology and precision agriculture and can consequently address food security issues.

## Introduction

Agricultural productivity is critically dependent on effective disease management strategies. Wheat rust, caused by various species of the Puccinia fungus, is one of the most devastating diseases affecting wheat crops worldwide. It can lead to significant yield losses, posing a threat to food security^1^. Traditional diagnostic methods for wheat rust involve manual inspection and expert analysis, which are time-consuming and not scalable for large agricultural fields^2,3^. Therefore, there is an urgent need for rapid, accurate, and scalable diagnostic tools. Deep learning (DL) methods have been successfully adapted for wheat rust image detection and classification achieving classification accuracy in the range of 96-99%^4,5,6^. Sharma et al. proposed a deep learning-based framework using multilayer perceptrons, achieving an accuracy of 96.24%^7^. Various deep learning approaches in plant disease detection, as demonstrated by Sladojevic et al.^8^, have been successfully employed for this purpose. Nigam et al.^9^ studied different computer vision models to study the highest accuracy when dealing with wheat rust data. Lu et al.^10^ made a significant breakthrough by modifying the traditional VGGNet deep learning architecture to create an in-field wheat disease detection system, which achieved an impressive accuracy of 97.95%. Notably, Singh et al.^11^ applied thermal imaging to tackle the problem, studying the results of Maximum Likelihood Estimations, Support Vector Machines, Neural Networks etc. Mi et al.^12^ proposed a model with a newer approach, with an embedding attention mechanism which outperformed ResNet.

Through the said DL methods, the most common and effective method highlighted is Transfer Learning^4^, wherein a large model is re-trained with domain-specific images. The model is more likely to generalize well and not overfit due to it being trained on a large dataset previously. This approach enables the efficient transfer of knowledge from related tasks, as deep learning (DL) models are capable of processing complex and large images, making them well-suited for analyzing high-resolution images^5^. However, these methods require a large amount of labeled training data and may not be suitable for unseen diseases. Furthermore, DL models are computationally expensive, which may be a limitation for some applications^13^. Li et al.^14^ performed semantic segmentation of wheat rust images, providing a unique path of research in the field highlighting the infections. Singhi et al.^6^ worked towards an integrated pretrained model to effectively detect wheat rust severity levels. Additionally, Maqsood et al.^15^ conducted a study and observed improved DL based detection results by using Super Resolution Generative Adversarial Networks (SRGANs), enhancing image-quality as well. Mohanty et al.^2^ conducted an extensive study on the variety of models available for transfer learning approaches for plant disease detection with a large dataset across 14 crop species and 26 diseases. All these studies simply classify the wheat image into their respective types or severity level. There is lack of an interactive system that offer a detailed understanding about the wheat rust images through question answering mechanism.

Visual Question Answering (VQA) models have gained significant attention in recent years as a means to enable machines to understand and respond to natural language queries about visual content. VQA models are designed to take an image and a question about the image as input and generate a relevant answer as output. Visual Question Answering (VQA)^16^ combines computer vision and natural language processing domains, enabling systems to answer questions about the content of an image. According to Antol et al.^17^, VQA models can be categorized into two main types: open-ended and multiple-choice. Open-ended VQA models generate a free-form answer, while multiple-choice models select an answer from a set of predefined options. Recent advances in VQA have been driven by the development of deep learning-based models, such as CNN-LSTM^18^and Bilinear Attention Networks^19^, which have achieved state-of-the-art performance on benchmark datasets such as VQA v2.0^20^. The integration of VQA systems with deep learning have the potential to revolutionize agricultural diagnostics by providing detailed and context-aware information about plant diseases directly from images. This can significantly aid farmers and agricultural professionals in making timely and informed decisions. By providing rapid and accurate diagnostics, such an approach can help mitigate the impact of diseases like wheat rust, improving crop yields and contributing to global food security.

In this paper, we present a visual question answering system for wheat rust detection and diagnostics by integrating deep learning with VQA. To enhance the performance of our VQA system, we implement Bootstrapping Language-Image Pre-training (BLIP) methods^16^. BLIP leverages large-scale pre-training on diverse image-text pairs to improve the model’s ability to understand complex visual and textual inputs. A dual attention mechanism is used to focus on relevant regions in the image and pertinent parts of the question simultaneously. We introduce a new WheatRustVQA dataset specifically designed for this task, which includes images of wheat plants annotated with questions and answers related to rust infection. Our dataset covers various environmental conditions, plant growth stages, and rust types, ensuring the robustness and generalizability of the models trained on it. The primary objectives of the paper include:

- Creation and application of a specialized WheatRustVQA dataset.
- Assessing the performance of the fine-tuned BLIP model in accurately answering questions about wheat rust symptoms and severity.
- Comparing our fine-tuned model and ChatGPT, highlighting the strengths of domain-specific training.

## Proposed work

**Fig. 1:**
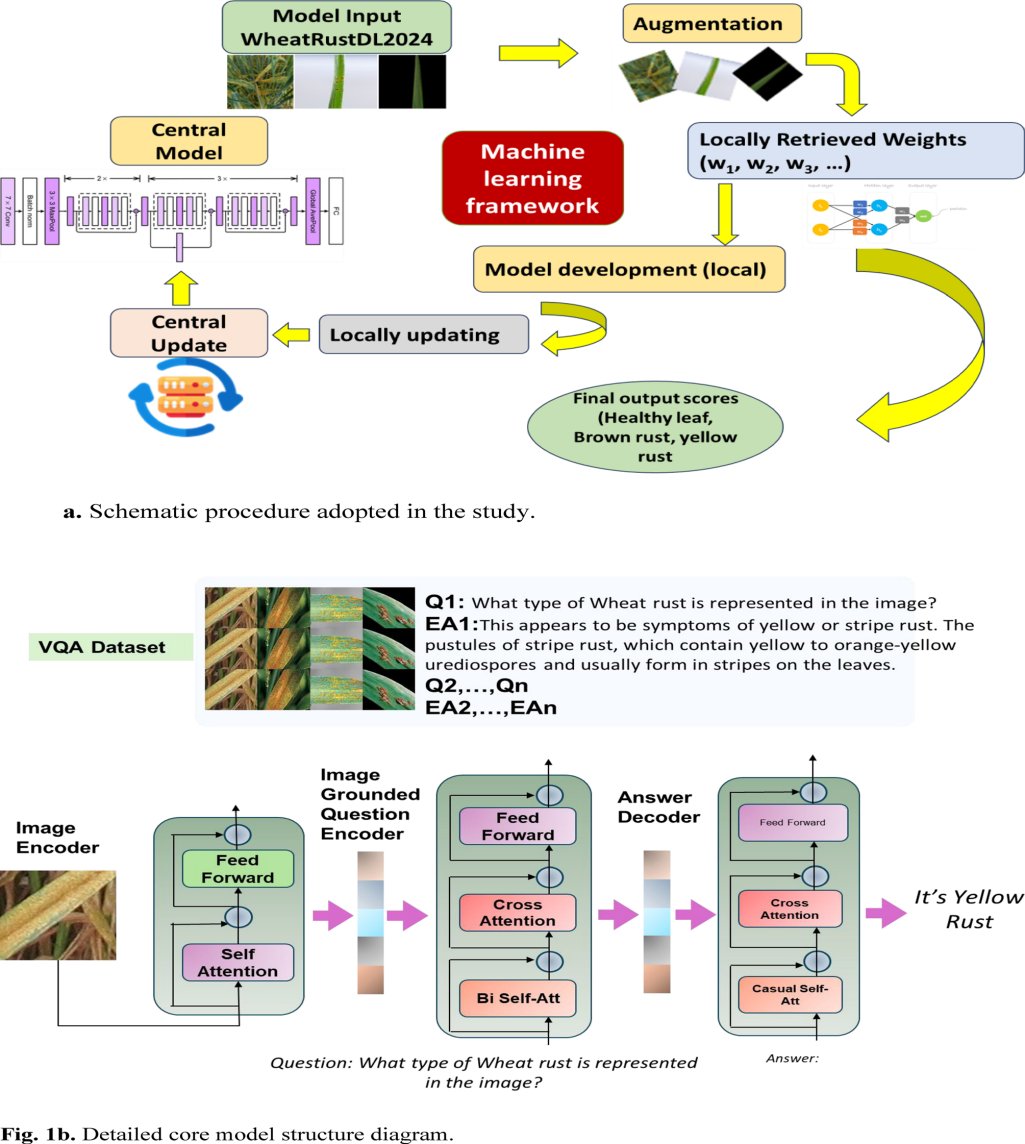
(a)Schematic procedure adopted in the study(b)Detailed core model structure diagram.

This study included an evaluation of state-of-the-art Convolution Neural Network (CNN) models for classification. Although previous literature exists on similar notes, the dataset used in this model is diverse, hence the model generalizes better. The models were evaluated, and the most accurate model was chosen as the central model. Alongside, text data was generated, and carefully vetted by industry professionals and agricultural research scientists, and our Visual Question Answering model was trained, creating a conversational interface for the detection of wheat rust. Figure 1a and b represent the schematic procedure and detailed core model structure adopted in the study.

We leverage Federated Learning to further enhance the classification system. Federated learning has advantages in terms of better data privacy with the flexibility to retain a part of the information off-premise on farms. It can also be used to seamlessly integrate machine learning models into drones for crop monitoring and analysis in real-time. It further ensures efficiency in handling data reduction to minimal transmission and storage. However, one apparent disadvantage of Federated Learning is that it increases the complexity of model training and deployment. In addition, model drift might occur, where decentralized models would eventually drift apart.

### Experimental setup

The Federated Learning as well as the Visual Question Answering Models were trained on the NVIDIA RTX™ A6000 GPU, with 48GB of memory.

### Novelty of the proposed approach

#### Dataset

A unique method of detecting wheat rust is through the use of a wide-ranging dataset. This represents a departure from previous investigations that utilized narrow datasets or only analyzed similar samples. For example, our WheatRustDL2024 dataset includes images showing different forms and degrees of rust infection on wheat leaves as well as healthy ones taken under varying lighting conditions, orientations, and resolutions among others. Such diversity in terms of these factors ensures exposure to many more real-world situations during training which in turn strengthens the system’s robustness, generality, and adaptability. With this kind of approach, it becomes possible for our model to detect accurately and classify wheat rust under any environmental condition thereby greatly contributing towards better disease control methods in agriculture.

#### Visual question answering

Additionally, in this study, the pioneering aspect of our research lies in the deployment of a state-of-the-art Visual Question Answering (VQA) system tailored specifically for wheat rust detection. VQA represents a cutting-edge intersection of computer vision and natural language processing, enabling machines to comprehend and respond to questions posed about images. Our VQA model is meticulously trained on a diverse dataset of wheat rust images, encompassing various rust severity levels, leaf orientations, and lighting conditions. By leveraging advanced deep learning architectures and attention mechanisms, our VQA system can accurately analyze images of rust-infected wheat leaves and provide detailed answers to questions regarding their condition. This innovative approach to VQA in the context of agricultural disease detection holds immense promise for facilitating rapid and accurate assessment of crop health, aiding farmers and agricultural experts in making informed decisions for disease management and crop protection.

### Study Data: images and text

**Fig. 2:**
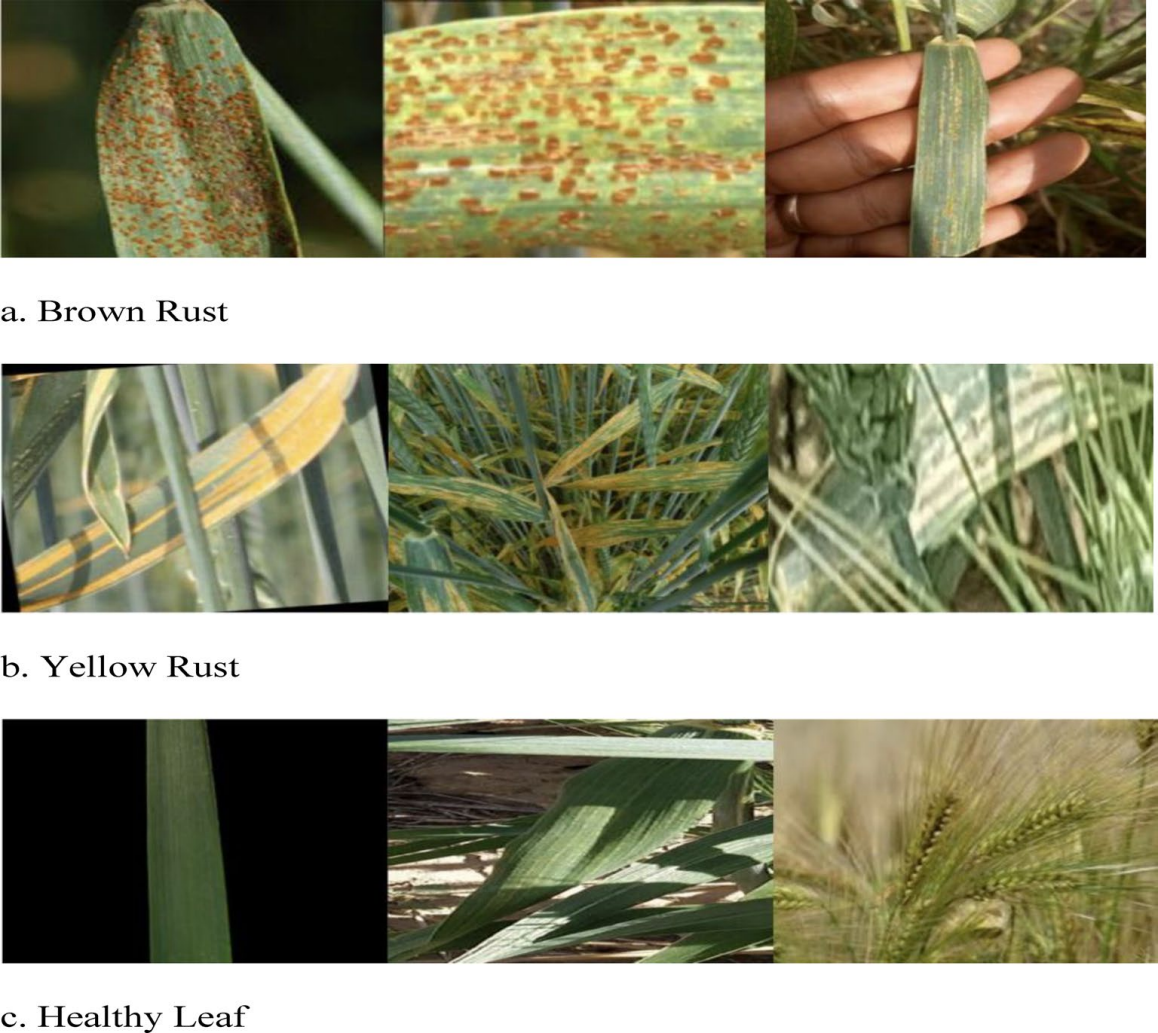
Sample images for each class of wheat leaves “brown rust”, “yellow rust”, “healthy leaves”.

The WheatRustDL2024 dataset (Fig. 2), a rigorously curated collection of images across the globe, was compiled to facilitate the development of robust machine learning models for wheat rust detection. The dataset also features data personally gathered by the study’s authors from various agricultural fields across India.

#### Image data

This comprehensive dataset comprises 7998 images, carefully selected from diverse sources, including field-captured images and existing datasets. The images exhibit a range of conditions, including varying lighting, orientations, and resolutions, thereby ensuring the representation of diverse scenarios under which a wheat leaf may be examined. The inclusion of images depicting healthy leaves, as well as those infected with stripe rust and brown rust, enables the development of a well-generalized model capable of accurately distinguishing between these conditions. The WheatRustDL2024 dataset (Table 1) provides a robust foundation for training and evaluating machine learning models, ultimately contributing to the advancement of wheat rust detection and diagnosis. The data was augmented by resizing it into images of a uniform dimension of 224 × 224. Along with normalization, image flips, random zooming and rotations were used to enhance the dataset.

**Table 1:** Different classes and description of the WheatRustDL2024 dataset.

Class	Description	Images	Images after Augmentation
Yellow Rust	Images of wheat plants with yellow rust, a common fungal disease caused by the fungus Puccinia striiformis. It is often found in regions with high humidity and cool temperatures.	2031	5673
Brown Rust	Images of wheat plants with brown rust, another fungal disease caused by the fungus Puccinia recondita. This rust species is typically found in warmer, drier regions and can be more difficult to control than yellow rust.	3591	7434
Healthy Leaf	Images of healthy wheat leaves, which serve as a baseline for comparison in the classification task. Healthy wheat leaves are typically green, glossy, and free of visible diseases or damage.	2477	10,357

#### Text data

The text data utilized in this study was gathered through a careful process involving the creation of relevant questions to the farmers and the subsequent generation of corresponding answers using ChatGPT. A template based on the generated answers was crafted, and this template was then factually corrected to ensure accuracy. The corrected descriptions were then forwarded to industry specialists, agricultural researchers and scientists who diligently vetted each description. The descriptions were specifically crafted for images depicting leaves with identical types and severity levels of rust. These descriptions served a crucial role in ground truthing each batch of images, facilitating the training and evaluation of a Visual Question-answering interface for wheat rust detection. This process was repeated systematically for each class of rust, ensuring creation of a comprehensive and reliable dataset for model development and testing.

#### Dataset Collection and Annotation

To create a robust VQA system for wheat rust detection, we developed a comprehensive dataset comprising high-resolution images of wheat plants. The dataset includes images captured under various environmental conditions and at different plant growth stages to ensure diversity and generalizability. Each image is annotated with multiple questions and corresponding answers related to the presence, type, and severity of rust infections.

#### Source

Images were collected from agricultural fields, research institutions, and publicly available datasets.

#### Diversity

The dataset includes 3 different types of wheat leaves (“yellow rust”, “brown rust”, “healthy leaves”). Samples for each class of wheat leaves are shown in Fig. 2.

#### Resolution

High-resolution images to capture fine details necessary for accurate diagnosis.

#### Questions

Each image is annotated with questions (Table 2) that could be asked by farmers or agricultural professionals, such as “Is there rust on this plant?“, “What type of rust is present?“, and “What is the potential impact on the yield?”

#### Answers

Corresponding answers are provided based on expert analysis and validated by multiple reviewers to ensure accuracy. The answers generated by ChatGPT 4 is also included for comparison purpose.

#### WheatRustVQA dataset

A total of 40 high-resolution images were collected, comprising 10 images each of yellow rust, brown rust, black rust-infested leaves, and healthy leaves (Table 3). For each of these images, 15 questions and their corresponding accurate answers were meticulously curated, resulting in an initial dataset of 600 data points, where each data point consists of an image, a question, and the subsequent answer. To augment the dataset and enhance model training, image augmentation techniques were applied, including horizontal and vertical flips of all images. This augmentation increased the dataset to a final size of 1800 image-text pairs, thus improving the robustness and generalizability of the model.

**Table 2:**
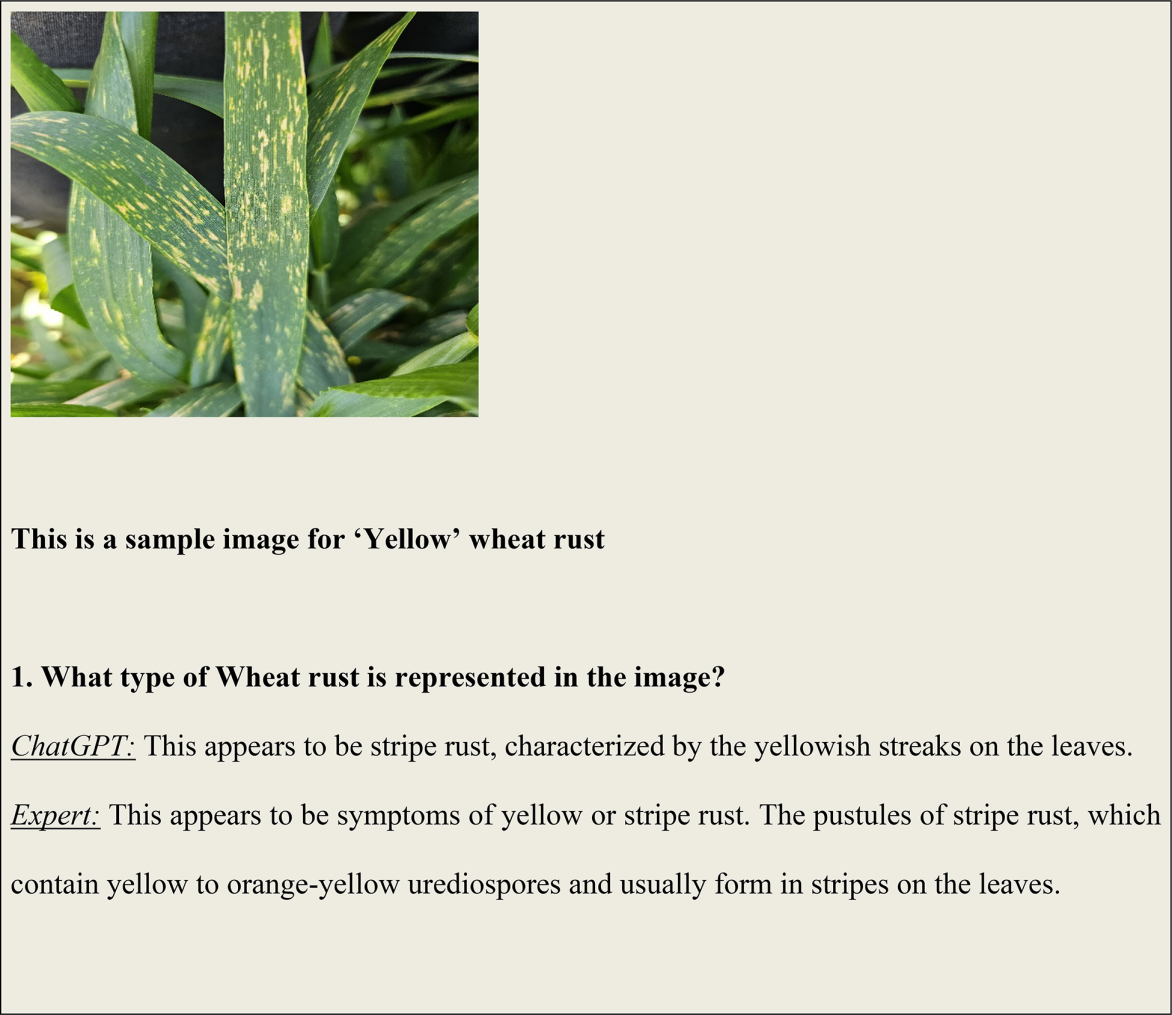

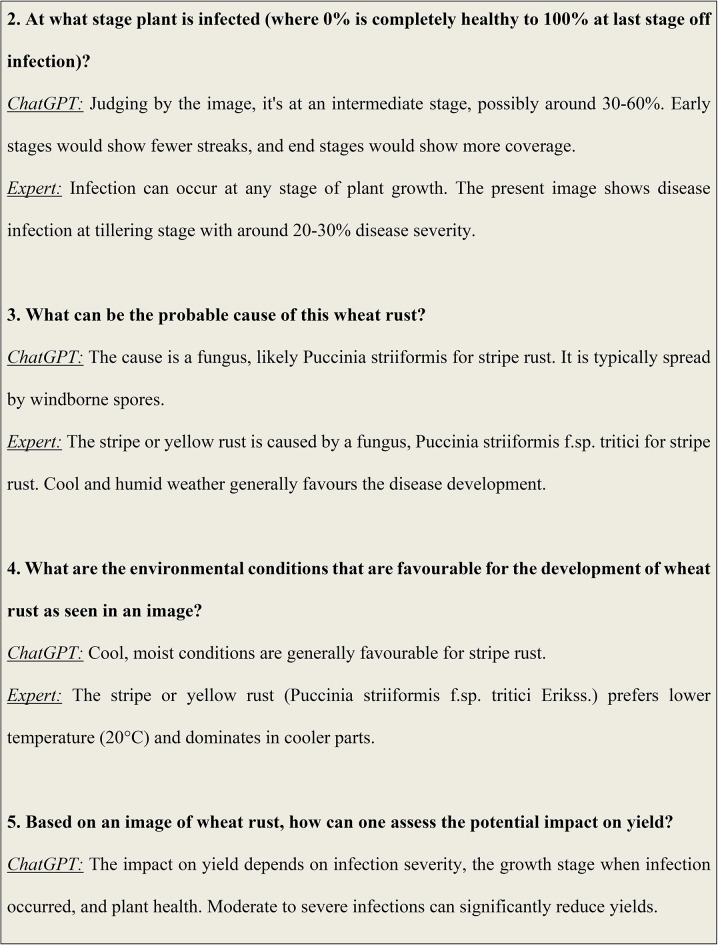

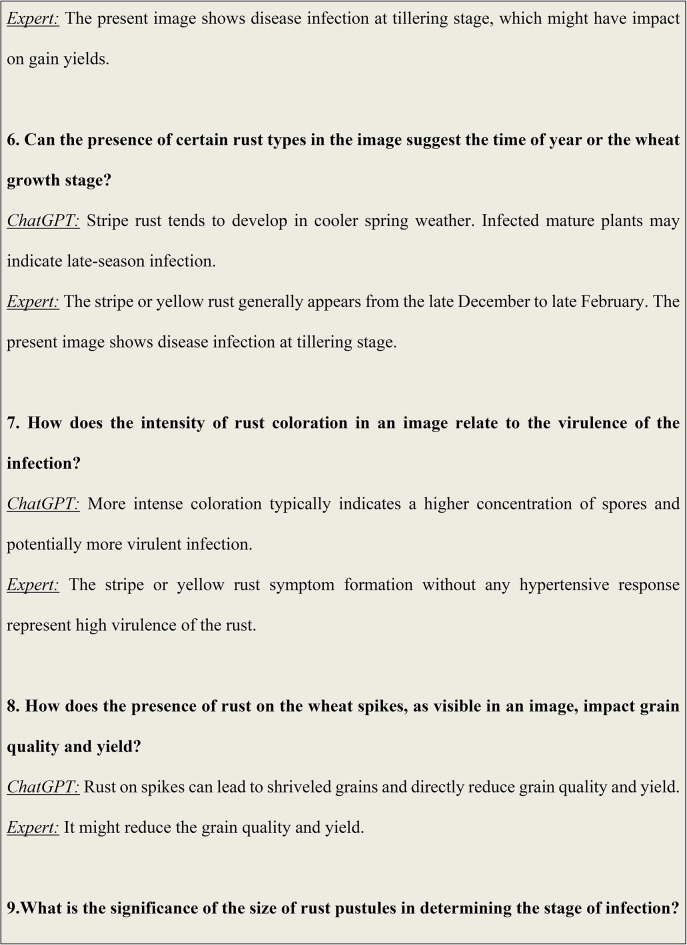

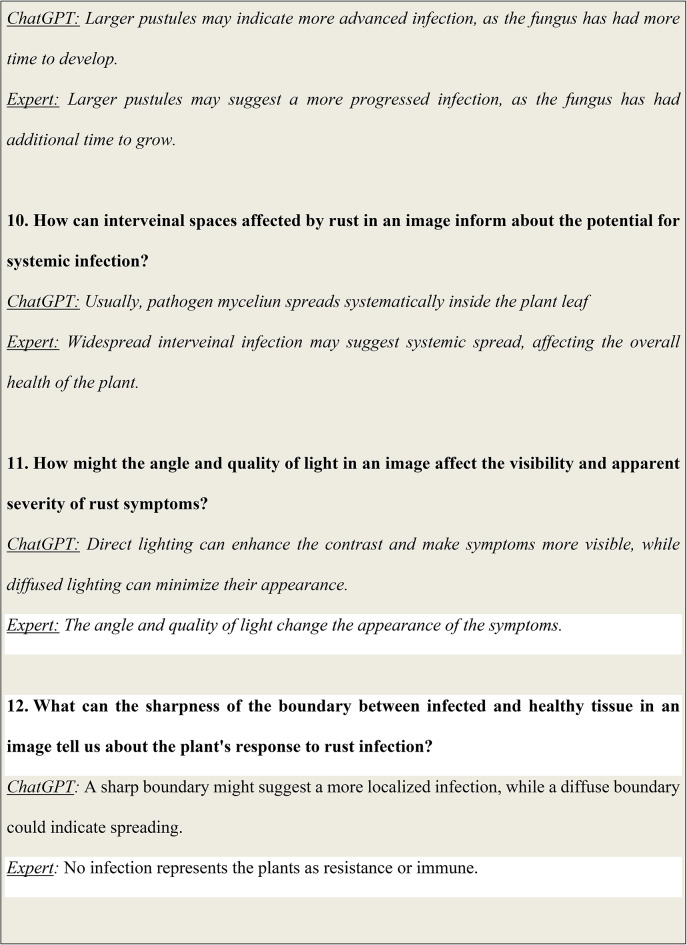

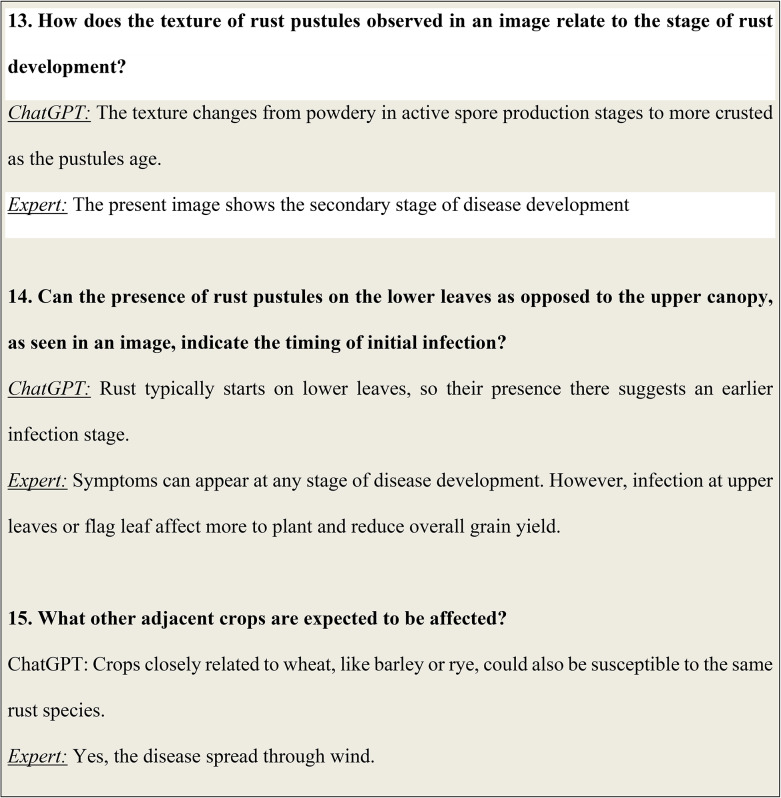
Deep learning models evaluated in the current study.

**Table 3:** Different classes and description of WheatRustVQA dataset.

Class	Description	Images	Images after Augmentation
Yellow Rust	Images of wheat plants with yellow rust, a common fungal disease caused by the fungus Puccinia striiformis. It is often found in regions with high humidity and cool temperatures.	10	30
Brown Rust	Images of wheat plants with brown rust, another fungal disease caused by the fungus Puccinia recondita. This rust species is typically found in warmer, drier regions and can be more difficult to control than yellow rust.	10	30
Black Rust	Images of wheat plants with black rust, a common fungal disease caused by the fungus Puccinia graminis. It is commonly found in cooler, moist regions and can be more difficult to control than yellow rust	10	30
Healthy Leaf	Images of healthy wheat leaves, which serve as a baseline for comparison in the classification task. Healthy wheat leaves are typically green, glossy, and free of visible diseases or damage.	10	30

### Central model

#### Transfer learning

Nigam et al.^9^conducted a study on the behavior of CNN models on wheat rust classification, A major conclusion of which was applying transfer learning on pretrained models gave better results compared to training a model from scratch on the dataset. Additionally, the training time in training from scratch was significantly higher, hence making it less feasible. Therefore, in this study, we work towards achieving higher accuracy by fine-tuning state-of-the-art computer vision models. The central model was chosen after a holistic evaluation of the state-of-the-CNN classification models. The unique characteristics of the dataset led to insights that contradicted findings from previous studies. The evaluated models included ResNet^21^, VGG^22^, EfficientNet^23^, and InceptionNet^24^, each of which was assessed based on metrics such as accuracy, precision, recall, and F1 scores. A brief description of the above models is given in Table 4. Following this evaluation, ResNet demonstrated superior performance across the given metrics and was thus chosen as the central model for the federated setting.

**Fine-tuning Process.**We finetuned our generalised state-of-the-art Convolutional Neural Network models to our particular use case of wheat leaf image classification into three prospective classes (Yellow Rust, Brown Rust, and Healthy Leaf). In this process, the WheatRustDL24 with 7998 images across 3 classes were used for finetuning with a split of 80% for train and 20% for test. In all models, the last layer was unfrozen, and a final classification layer was added to three classes to suit the scope of our study. The optimiser used in the finetuning process was Stochastic Gradient Descent, with a learning rate of 0.001. Additionally, a momentum of 0.85 was applied for optimisation and a weight decay of 1e^[-5^ was applied for regularisation. The loss function used in this process was cross entropy across all state-of-the-art models.

**Table 4:** Deep learning models evaluated in the current study.

Model	Architecture	Description	Layers		Parameters	Optimizer	Reference
ResNet	Deep CNN with residual connections	Uses residual connections to avoid vanishing or exploding gradients	152	23.5 million		Adam	^21^
Inception Net	CNN with inception modules	Employs inception modules for multi-scale feature extraction	22	22.8 million		RMSprop	^24^
VGGNet	Deep CNN with varying depths	Known for its uniform architecture and deep layer configurations.	16	138 million		Stochastic Gradient Descent	^22^
EfficientNet	Neural network with compound scaling and model efficiency	Utilizes compound scaling for balancing model size and performance	7	5.3 million		Adam	^23^

Subsequently, the model was federated using FLOWER^25^, a framework that integrates mechanisms for detecting and mitigating Byzantine attacks. This incorporation ensures the integrity of model aggregation in decentralized environments, further enhancing the robustness and reliability of the federated learning system.

##### Federating strategy

Federated averaging, integral to our study’s federated learning approach, facilitates collaborative model training while respecting data privacy. Table a represents the federated average algorithm used in this study. Each device, such as those monitoring wheat rust, refines a local model using its respective data and sends model updates, not raw data, to a central server. The server aggregates these updates through averaging, enhancing the global model without exposing individual data. This process ensures robust learning across diverse data sources, crucial for our agricultural application’s privacy and data security considerations. Federated averaging is a distributed optimization algorithm that enables multiple devices to collaboratively train a model while preserving data privacy. The mathematical representation of FedAvg can be expressed as Eq. (1)

$$\:FedAvg=\left(1-\alpha\:\right)\times\:\alpha\:\left(localmodel\right)\left(1\right)$$

where α is a hyperparameter which signifies the weight of the local updates, signifying the emphasis of the local updates on the central model updating on the server. In this study, α has been chosen proportional to the number of samples in the dataset updated. Therefore, for a local client accessing the model, having a dataset of $\:N$ images, $\:\alpha\:\propto\:N$.

**Fig. a:**
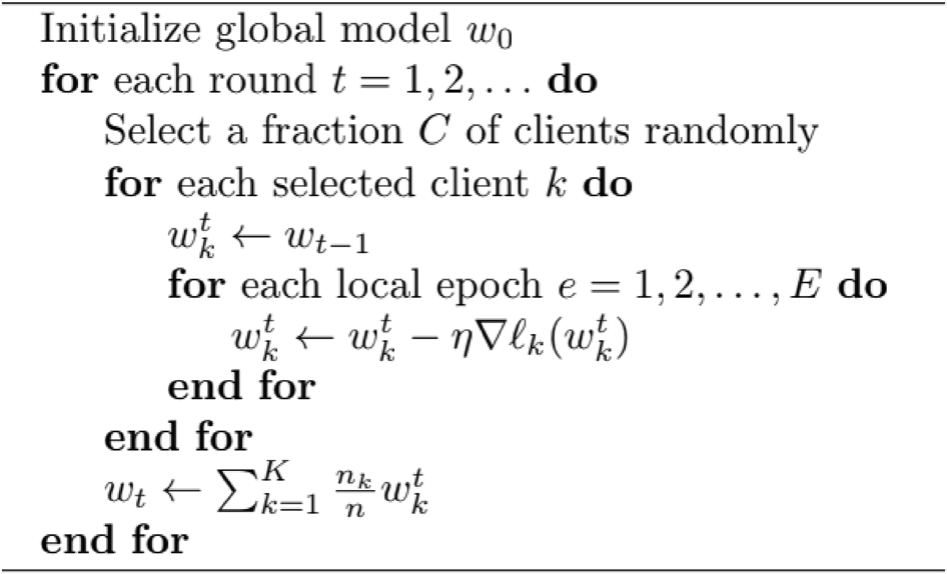
Accuracy and Loss curves associated with EfficientNet-B0 & B1.

### VQA Model Architecture

**Fig. 3:**
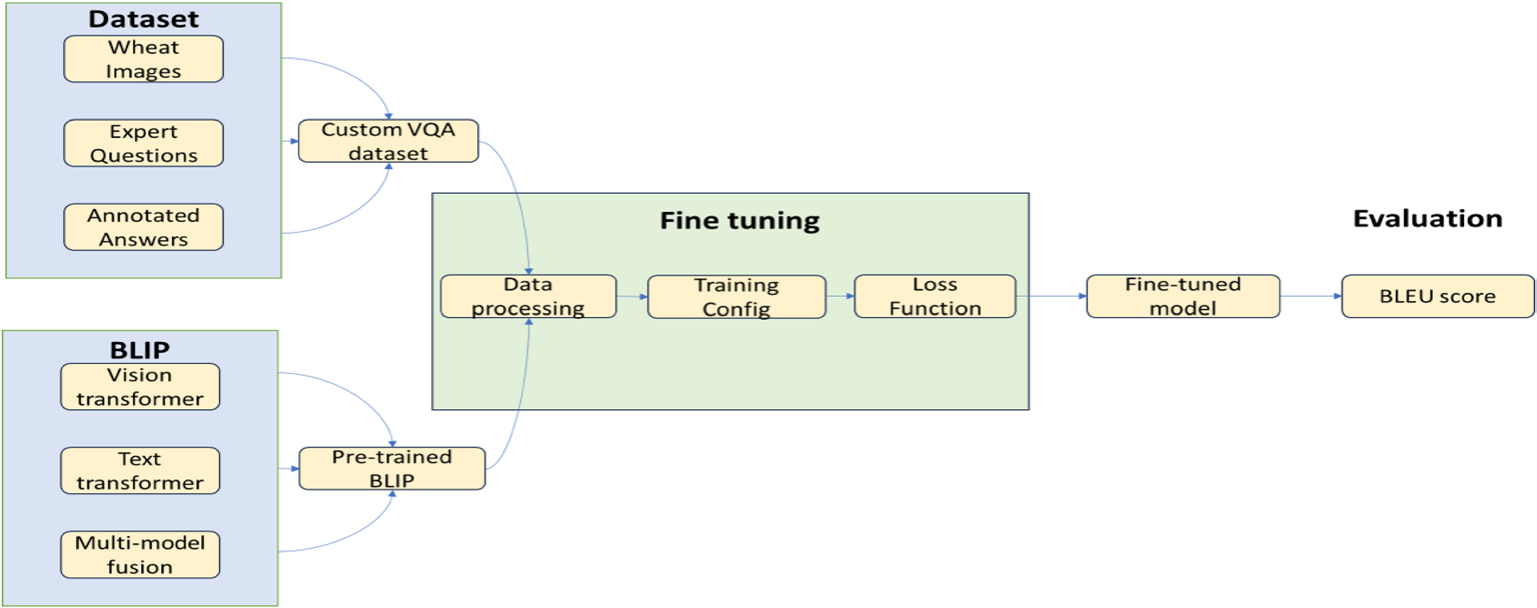
VQA Pipeline Using Fine-tuned BLIP for Wheat Rust Diagnostics.

Our proposed method for visual question answering in agricultural disease diagnostics, specifically for wheat rust, leverages the power of the BLIP (Bootstrapping Language-Image Pre-training)^16^ model. We utilize the BLIP model as our foundation due to its strong performance in vision-language tasks. BLIP, is a unified vision-language model that excels in various tasks, including visual question answering. The model’s architecture consists of a vision transformer (ViT) for image encoding, a text transformer for language understanding and generation, and a multimodal fusion module that combines visual and textual features (Fig. 3). This architecture allows for effective joint reasoning over image and text inputs, making it suitable for our agricultural disease diagnostics task.

**Fine-tuning Process.** We fine-tuned the pre-trained BLIP model on our custom wheat rust VQA dataset. The fine-tuning process involves data preprocessing where the image is resized and normalized to match BLIP’s input requirements. It also involves tokenizing questions and answers using BLIP’s text encoder. We use AdamW with a learning rate of 10^−5^, batch size: 32. We employ a combination of cross-entropy loss for answer prediction and contrastive loss to enhance the alignment between visual and textual features. Initially we freeze the vision transformer to preserve learned visual features and gradually unfreeze layers, allowing for domain-specific visual feature adaptation. By fine-tuning BLIP on our specialized wheat rust VQA dataset, we aim to create a model that can accurately interpret visual cues in wheat plant images and provide relevant answers to diagnostic questions, thereby assisting in the identification and assessment of wheat rust infections.

In this finetuning process, the model textual outputs for the WheatRustVQA images and questions dataset were trained against the ground truth answers. The dataset was split into train (1750 tuples) and test (50 tuples) due to the dataset being narrow.

#### Benchmarking

We benchmark our VQA model using BLEU (Bilingual Evaluation Understudy) score. The BLEU score measures the similarity between machine-generated and human-written text, by calculating the precision of n-grams in generated text compared to the reference text. The score ranges from 0 (no similarity) to 1 (perfect match).

 Para ID=“Par13”>

## Results

### Model evaluation results

#### Central Model

The study commenced with a thorough analysis of state-of-the-art deep learning models to choose as the central model. This initial study was done on various IncpeptionNet v3, VGG 16, ResNet 50 and 152, and EfficientNet B0, B1, B2, B3 models. The accuracy of these models on the raw dataset and the main dataset after augmentations, WheatRustDL2024, are given in Table 5.

**Table 5:** Accuracy of deep learning model on the raw dataset.

Model	Accuracy on Raw Data		Accuracy on WheatRustDL2024	
	Training	Testing	Training	Testing
InceptionNet v3	67.68	67.98	63.5	69.73
VGG 16	88.30	88.81	86.21	88.36
ResNet 50	98.67	95.81	99.13	97.69
ResNet 152	98.41	94.68	98.72	97.62
EfficientNet B0	96.50	95.68	94.64	95.76
EfficientNet B1	95.26	94.81	94.21	95.76
EfficientNet B2	95.09	94.18	93.86	95.38
EfficientNet B3	67.27	75.38	94.30	95.03

**Fig. 4:**
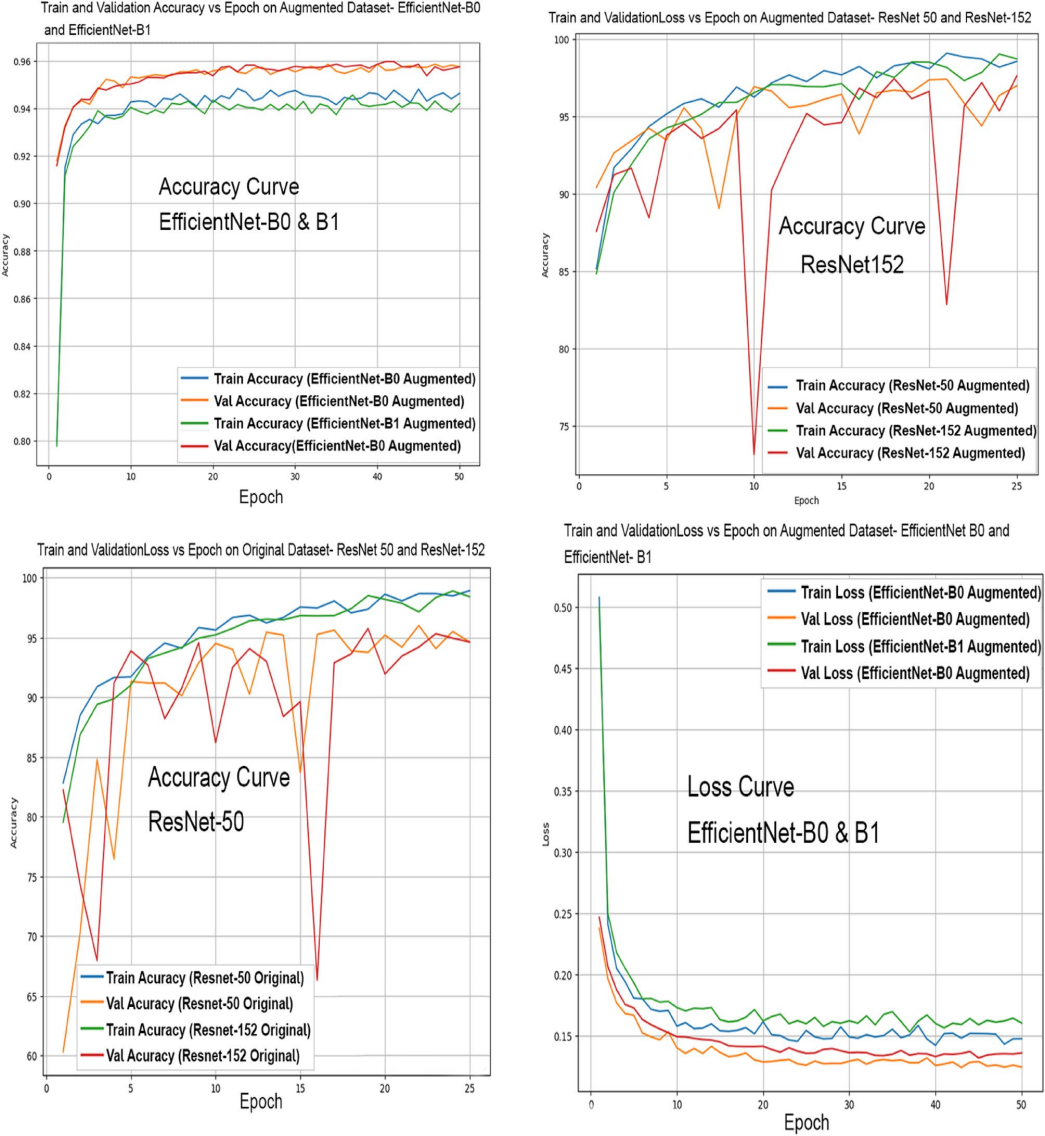
Accuracy and loss curves for ResNet 50 & 152

**Fig. 5:**
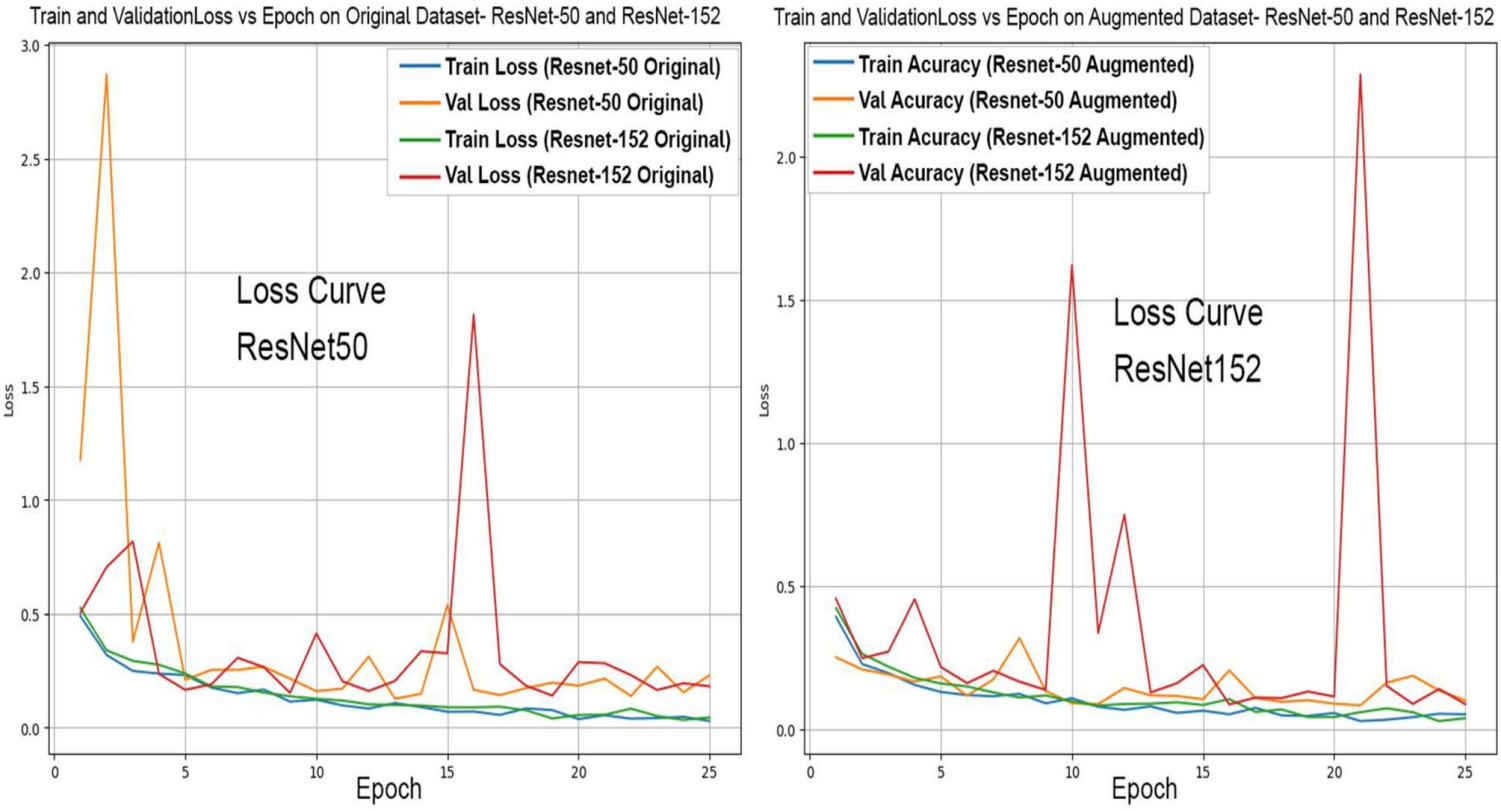
Sample images for each class of wheat leaves “brown rust”, “yellow rust”, “healthy leaves”.

For illustration, Figs. 4 and 5 represent the accuracy and loss curves associated with ResNet 50, 52 and EfficeintNet B0, B1. Initially, the model was trained on raw data. The InceptionNet v3 model achieves a training accuracy of 67.68% and a validation accuracy of 67.98%. The corresponding values for VGG 16, ResNet 50, ResNet 152, The EfficientNet B0, EfficientNet B1, EfficientNet B2, EfficientNet B3 models are [88.30%, 88.81%], [98.67%, 95.81%], [98.41%, 94.68%], [96.50%, 95.68%], [95.26%, 94.81%], [95.09%, 94.18%]. [67.27%, 75.38%] respectively. Most models outperformed on the main dataset with preprocessing augmentation methods. Notably, the ResNet families outperformed with an accuracy increase of 4–5% each, however even more notably, EfficientNet B3 outperformed the previously trained model with an accuracy increase of almost 20%. The accuracy of the model trained on raw data is generally lower than the accuracy when trained on the WheatRustDL2024 dataset. This difference can be attributed to the complexity of the raw data and the challenges in accurately labelling and processing it. The models perform better on the WheatRustDL2024 dataset, which may have been preprocessed or labelled more accurately, leading to improved model performance. Therefore, the main criterion for a model to have a high accuracy is its ability to generalize well, which had a low benchmark for models trained on raw data and a relatively higher benchmark for models trained on augmented data. Owing to the high accuracy of ResNet-50 on WheatRustDL24, it was chosen as the central model in the federated environment. This model now has downloadable weights and is constantly updated with federated averaging.

### Analysis results of visual question answering

**Fig. 6:**
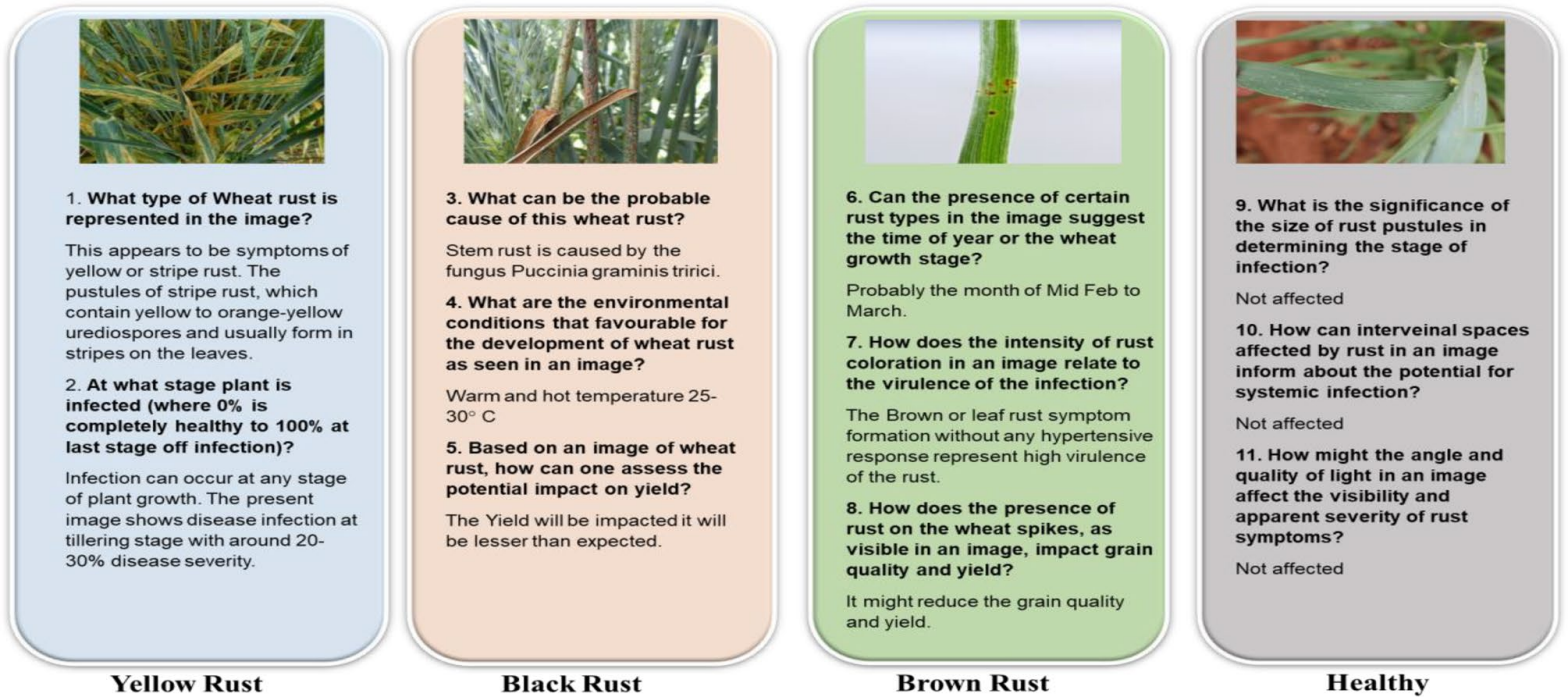
An illustration of some of the questions and answers used in the VQA model.

**Fig. 7:**
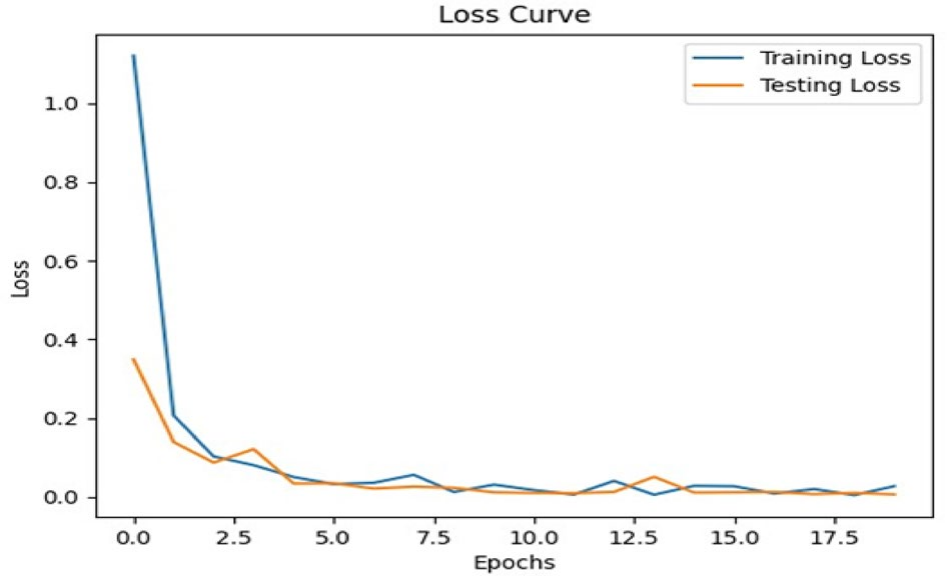
The loss curve for finetuning the VQA model.

Figure 6 illustrates some of the questions and answers used in the VQA model. The fine-tuned VQA model gave a training average language modelling loss of 0.0071 on our test dataset of images and question-answers, and a test BLEU score of 0.6235. A snippet from our model testing is presented in Fig. 7 by inputting a random image of a wheat leaf and feeding prompts requesting more details on the same. To minimize the possibility of overfitting, image augmentation was applied to increase the dataset size. Keeping the narrow dataset in mind, the decision of fine-tuning was taken, and these results were tested on a unique dataset of images. The Loss Curve for the proposed VQA model is presented in Fig. 7, plotting the total loss across the dataset after each epoch, whereas Fig. 6 illustrates some of questions and answers used in the VQA model.

## Discussions

Wheat rust detection remains a crucial task to address. Stem rusts are a significant threat to wheat production. Secondly, as global food security becomes increasingly important, ensuring the health of cereal crops like wheat is essential. Accurate detection allows growers to make timely management decisions, preventing further spread and minimizing yield losses. Therefore, in this study, we provided a federated setting for a deep learning model and a Visual Question Answering concept that would transcend the realm of the ongoing research towards tackling wheat rust.

### Federated Deep Learning with better accuracy using varied datasets

This study commences with a brief study of the state-of-the-art convolutional neural network models. We achieved notable accuracies in the ResNet and EfficientNet architectures. Previous studies on wheat rust identification^26,27^ had a narrow range of classes and a highly specific dataset. Nigam et al.^9^ conducted a thorough analysis with a larger dataset, although it was not generalised, and they achieved model accuracies in the range of 95–96% on EfficientNet architectures, with the highest recorded being 95.76%, while they achieved 95.69% on ResNet-152. Additionally, their findings of the accuracy for VGGNet 19 was at 90.89%, similar the accuracy of VGGNet 16 was 88.36% in our study, a lighter model with 16 layers compared to the 19 in VGGNet 19.

Across the Deep Learning applications in wheat rust detection, Chang et al.^28^ proposed an improved Dense Net architecture with an added attention network on the Whet Common Disease Dataset, Wheat Rusts Dataset and the Common Poaceae DIsease Dataset, achieving accuracies of 98.32%, 96.31% and 95.00% respectively. Adem et al.^29^ conducted a comparative analysis of deep learning parameters such as layers, activation functions and optimisers, concluding that a 5-layer CNN model using activation function Leaky ReLU using the Nadam optimiser gave the highest accuracy of all in their study on a custom dataset, at 97.36%, an improvement from 90.87% from their default parameters. Liu et al.^30^ worked towards leaf segmentation, creating a mobile-based deep-learning model that achieved a 98.65% Mean Intersection over Union (MIoU) in segmentation. Khan et al.^31^ worked towards implementing a Convolutional Neural Network on a dataset of 407 images of wheat rust, aiming to further integrate this model into edge devices. They achieved an accuracy of 98.77% on their test. Nigam et al.^32^ added convolution block attention mechanisms to the EfficientNet B0 model to create a custom deep learning architecture. The custom model of this study achieved an accuracy of 98.70% on their test dataset and an F1 score of 98%. Broadly, the results of the high performance of deep learning architectures could be well substantiated by the previous analysis of deep learning architectures catered towards the classification of wheat rust, with our study emphasizing a more generalized dataset with varied conditions in place.

Such higher efficiency associated with federated learning as observed in this study is very much in line with previous studies on disease detection. Federated Learning is a natural step forward in the paradigm of deep learning. It is one of the few efficient models that account for privacy preservation and accessibility. It has already seen widespread adoption. It is widely used for disease detection while keeping sensitive patient data private. For instance, a federated learning approach was used to predict hospitalizations for cardiac events using a binary classification supervised model^33^. In the field of advertising, Facebook has employed federated learning to rebuild its ad system by running algorithms locally on phones to find out what ads a user finds interesting, respecting user privacy^34^.

Along with the general industry applications, Federated Learning has found applications in plant disease identification. Kabala et al.^35^ conducted a study analysing computer vision models and adopting a federated setting with Federated Average (FedAvg) as the strategy. They additionally tested Vision Transformers (ViTs), which is not as relevant for this study, as we deal with data consisting of fewer background features, making a transformer layer redundant. Hari et al.^36^ on similar notes proposed an improved federated learning model for plant leaf disease detection by analysing FedAvg and FedAdam.

Federated learning is a move forward toward enhancing the detection and classification of disease in agriculture since it allows the use of decentralized data and achieves better performance in models. Past studies in agriculture have been aggressively supporting this idea since Federated Learning addresses data privacy^35–38^. These studies have therefore proven the feasibility of Federated Learning for these agri-tasks: crop yield prediction, and disease diagnosis. Our research raised the question of data privacy in agricultural contexts and underlined the necessity for data in agriculture to be processed securely and decentralized. Moreover, our study defined the versatility of the application of Federated Learning at the edge device to offer real-time processing of data that reflects farming activities speedily. Our model classifies a general dataset and hence is used in strong and precise detection of diseases across different agricultural settings. Our work differs from the federated learning studies mentioned above concerning their application in agricultural-related issues, because the area of study concentrated on wheat rust disease, which affected most wheat plants worldwide. The study, thus, attempts to examine the applicability of Federated Learning in detecting wheat rust to make a contribution towards better and more efficient disease management strategies in the agricultural sector.

To the best of our knowledge, Federated Learning has not been implemented on wheat rust detection, where multiple agricultural bodies could collaborate and enhance a centralised architecture using their respective diverse datasets which also preserves their data privacy. Additionally, its lightweight nature makes it more suitable for integration with devices such as smartphones.

In this study, we proposed a federated central model that improves the strategy of Federated Averaging (FedAvg) naturally as it gives a higher weightage to a client that provides a larger dataset. Further work in this field could be towards another federating strategy or a simulation where the dataset is divided into multiple clients to analyse an optimal strategy. Apart from Federated Averaging, several other federated learning strategies have been proposed to improve the performance and efficiency of distributed machine learning. For instance, Federated Stochastic Gradient Descent (FedSGD) has been shown to converge faster than FedAvg in certain scenarios^39^. Another approach is Federated Proximal (FedProx), which incorporates a proximal term to handle heterogeneous data distributions across clients^40^. Additionally, Federated Multi-Task Learning (FedMTL) has been proposed to learn multiple related tasks simultaneously, leveraging shared knowledge across tasks^41^. These alternative strategies offer different trade-offs between communication efficiency, model accuracy, and robustness to non-IID data.

Another interesting field of study in Federated Learning lies in the selection of topology. In this study, we work with a centralised model that a client can access locally. Multiple conceptual topologies exist in Federated Learning, with the standard federated learning topology being the main aim of the study, creating a parent model. On other trajectories, experimentation with other topologies, such as the Star Topology, Tree Topology, Decentralised Topology etc. may also be a field of study in efficient wheat rust detection.

### Multi-modal interactive interfaces

Interactive AI systems have revolutionized various industries by enabling dynamic human-machine interactions. These systems leverage natural language processing (NLP) and machine learning techniques to understand and respond to user queries, commands, and requests in real-time. They are deployed in virtual assistants, customer service chatbots, educational platforms, and smart home devices, enhancing user experiences and streamlining tasks. Specifically, Visual Question Answering (VQA) models have emerged as a powerful application of interactive AI, allowing users to pose questions about visual content and receive accurate responses.

The importance of the VQA approach alongside federated classification in this study is prominently highlighted by Ryo et al.^42^, who emphasized the need for incorporating social and human opinions into Artificial Intelligence. Their studies showed an abundance of research using CNN for image analysis and recurrent neural networks for time-series analysis. They suggest the use of multi-model AI, one taking data with different features such as image, audio, text, smell, etc. Therefore, VQA models pave the way for a new field of modelling as well, combining image features and text features, while being trained on data. Plant disease detection has seen the application of Natural Language Processing, with Guofeng et al.^43^ proposing a fine-tuned BERT for question classification of common crop diseases question answering systems, and Jain et al.^44^ proposing an agricultural question answering system. These are purely NLP applications, and do not take a step towards integrating image data, achieving multimodality.

Arora et al.^45^ introduced Long Short-Term Memory (LSTM) Recurrent Neural Network architectures, and also applying a Convolutional Neural Network for classification, therefore achieving multimodality, which, however, is not integrated, unlike VQA models. Along similar lines, LSTMs were also applied to agriculture by Shang et al.^46^ along with CNN models. Towards fruit tree disease estimation and decision making for further steps, Lan et al.^47^ created a Visual Question Answering model using Tucker decomposition to fuse image features with the subsequent text features. Similarly, Lu et al.^48^ worked towards a multimodal interface in agricultural disease detection by applying multimodal transformer architectures, subsequently creating a question-answering model.

In the context of this study, Generalisability refers to the model being able to correctly navigate through unseen data, similar to the kind of data the model is trained upon. In the context of this study, our dataset is generalised in the sense, that it consists of images of crops with similar diseases with different camera angles and lighting conditions, as well as varying levels of disease severity. Therefore, the model is trained upon different conditions in which a farmer or sensor might capture the image of a wheat leaf, including diverse environmental factors and image qualities. This is important since this allows the application of this model in different agricultural settings, including small-scale farms and large-scale industrial plants, where the model can be used to detect wheat rust in a variety of scenarios. However, to reinstate the scope of this study, generalisability does not concretely refer to generalisability across domains, such as (but not limited to) plant diseases other than wheat rust, or different types of crops. The models in this study are trained on a wheat-rust-specific dataset according to the scope of the study. Therefore, the models are generalisable within the scope of wheat rust detection specifically, and are expected to perform well on detecting wheat rust in various images, but may not perform well on detecting other types of plant diseases.

Additionally, Robustness refers to the model’s ability to maintain its performance even when the input data is noisy, incomplete or corrupted. In the context of this study, our model is designed to be robust to variations in image quality, such as different resolutions and compression levels. This is important because, in real-world agricultural settings, images of wheat leaves may be captured under varying conditions, such as with different camera and sensor qualities. By training our model on a dataset that includes images with these variations, we ensure that it can accurately detect wheat rust even when the input data is slightly corrupted. However, to clarify the scope of this study, robustness in this context does not imply that the model is completely robust to all possible types of noise or corruption, but rather that it is robust to the types of variations that are commonly encountered in agricultural settings, due to a variation in the quality of equipment. Specifically, our model is robust to variations in image quality that are typical of images captured in the field, but it may not be robust to more extreme types of noise or corruption that are not commonly encountered in agricultural settings.

This study explores a newer field of research in wheat rust detection. Additionally, with the Federated Learning interface, we explore VQA models to create an interactive interface for further research. This initial model was trained using 15 questions and answers for 40 images across four classes, yellow rust, brown rust, and healthy leaves, creating a custom dataset of 600 data points. VQA models can be broadly classified by the type of answers they process. They are generally models that process multiple choice answer type, short answer type and long answer type labels. The scope for a multiple-choice model in this particular field is less, although it would increase model objectivity, and correspondingly its accuracy. Therefore, given a suitable problem statement in the field of wheat rust detection, such a model can be applied to an appropriate dataset. Alternatively, there also exist models which work with short answers. There exists an abundance of such models.

## Conclusions

This study introduces a novel approach to wheat rust diagnostics by integrating deep learning-based visual question answering (VQA) techniques. We established a baseline for this critical agricultural application by fine-tuning the BLIP model on our newly created WheatRustVQA dataset. Our research demonstrates the potential of VQA systems in plant pathology and provides valuable insights through a comparative analysis with ChatGPT. This work has significant implications for enhancing disease diagnostic capabilities in agriculture through image-based question answering. It also paves the way for more accessible expert-level diagnostics in regions with limited access to plant pathologists.

We show the performance of our system and report the standard VQA metrics that serve as baseline for wheat rust VQA. Our results indicate that the integration of BLIP methods with VQA models significantly enhances diagnostic accuracy, reducing the need for expert intervention. The integration of deep learning with VQA for agricultural disease diagnostics represents a significant advancement in precision agriculture. By providing rapid and accurate diagnostics, our approach can help mitigate the impact of diseases like wheat rust, improving crop yields and contributing to global food security. This paper lays the groundwork for future research and applications in plant pathology, highlighting the potential of deep learning and VQA in transforming agricultural practices.

While our results are promising, limitations include the need for further validation in varied field conditions and potential challenges in generalizing to other crop diseases. Future work should focus on expanding the dataset, exploring multi-disease models, and integrating this technology into mobile applications for real-time field use. In conclusion, this research lays a solid foundation for the application of AI in agricultural disease diagnostics. As we continue to refine these models and methodologies, the integration of VQA in agriculture promises to play a crucial role in addressing plant disease challenges and contributing to global food security.

## Data Availability

• This study introduces a Federated Learning and a Visual Question-Answering model. These models are available online on this study’s GitHub (https://github.com/aknnvt/FL-VQA-in-Wheat-Rust). • The custom datasets curated for this study, WheatRustDL2024 (https://bitspilaniac-my.sharepoint.com/:f:/g/personal/f20212378_pilani_bits-pilani_ac_in/EvwjsY_JT4FIu7ZTU8zyXOMB8Ywk4OXgO6LYwTk8dOiN_Q? e=45eTOD) and WheatRustVQA (https://docs.google.com/document/d/1EvVdrMi7W-JZkeeePEkNmVIEJ1dn7eln/edit? usp=sharing&ouid=114090611032812705334&rtpof=true&sd=true), are available for public use. Additionally, the images in WheatRustVQA (https://drive.google.com/drive/folders/1izs5ZVmi9V__ixk4ODiJAyachAulP3RL? usp=drive_link). The raw version of the answers can be found on the GitHub repository.
